# Cross-population tongue image features and tongue coating microbiome changes in the evolution of colorectal cancer

**DOI:** 10.3389/fmicb.2025.1442732

**Published:** 2025-02-12

**Authors:** Fang Liu, Dan Su, Xing Shi, Shu-min Xu, Yu-kun Dong, Zhi Li, Bo Cao, Dong-lin Ren

**Affiliations:** ^1^Department of Coloproctology, The First Affiliated Hospital of Guizhou University of Chinese Medicine, Guiyang, China; ^2^Department of Coloproctology, The Sixth Affiliated Hospital, Sun Yat-sen University, Guangzhou, China; ^3^Guangdong Provincial Key Laboratory of Colorectal and Pelvic Floor Diseases, The Sixth Affiliated Hospital, Sun Yat-sen University, Guangzhou, China; ^4^The First Clinical Medical School, Guizhou University of Traditional Chinese Medicine, Guiyang, China

**Keywords:** tongue coating microbiome, tongue image features, colorectal cancer, colorectal adenoma, traditional Chinese medicine, 16S rRNA sequencing, high-throughput sequencing

## Abstract

**Introduction:**

Tongue diagnosis, a cornerstone of Traditional Chinese Medicine (TCM), relies significantly on the assessment of tongue coating, which is used to evaluate Zang-fu organ functions, qi and blood dynamics, and the influence of pathogenic factors. This diagnostic method is integral to disease diagnosis and treatment in TCM. Recent research suggests a strong correlation between the characteristics of tongue coating and its microbial composition. These microbial variations may influence the formation and changes in tongue coating and are potentially linked to the progression of specific diseases. However, comprehensive research on the association between tongue coating, its microorganisms, and colorectal cancer (CRC) is limited. Notably, the quantitative aspects of tongue diagnosis and the microbial diversity in tongue coatings across different stages of colorectal cancer (from healthy individuals to colorectal adenoma (CRA) and CRC patients) are yet to be fully elucidated. By studying the cross-population characteristics of tongue image and tongue coating microorganisms during the evolution of colorectal cancer, the differences of tongue image characteristics and tongue coating microorganisms among different populations were further evaluated, providing references for early screening, diagnosis and treatment of colorectal cancer.

**Methods:**

The tongue image features of the subjects were collected by DS01-B tongue surface information collection system, mainly including tongue quality and tongue coating, and the tongue image was quantitatively analyzed by color space Lab value. The microbial characteristics of tongue coating were detected by high-throughput sequencing (16SrRNA amplicon sequencing). All subjects came from the patients in the Sixth Affiliated Hospital of Sun Yat-sen University and recruited volunteers (divided into health group, CRA group and CRC group), and obtained the ethical approval of the Sixth Affiliated Hospital of Sun Yat-sen University (ethical batch number: 2021ZSLYEC-328).

**Results:**

A total of 377 subjects were recruited in this study, including 56 healthy subjects, 65 colorectal adenomas and 256 colorectal cancer patients. The results showed that: in terms of texture of fur, the “thick fur” was a significant statistical difference (*p* < 0.05) in the 3 groups. In addition, there was also a statistical difference in “greasy fur” and “peeled fur” among the 3 groups (*p* < 0.05). Lab quantitative analysis of tongue color and fur color: The results showed that the L value of tongue color in healthy group was significantly different from that in CRA group and CRC group (*p* < 0.01), but there was no significant difference between CRA group and CRC group (*p* > 0.05). Tongue coating microorganisms, there was no significant difference in the richness and diversity of the three groups of subjects (*p* > 0.05). There were 296 species in the three groups, accounting for 44.65%, and the species in colorectal cancer population was the most, reaching 502. From the differences in community composition among the three groups, it was found that there were certain differences in bacterial community composition between healthy people, CRA and CRC, and the differences became more and more obvious with the development of the disease.

**Conclusion:**

This study revealed the specific cross-population tongue image characteristics and the specificity of tongue coating microorganisms in the evolution of CRC, providing new research ideas for early screening, early diagnosis, mechanism exploration, prevention and treatment of colorectal cancer.

## Introduction

The incidence of colorectal cancer (CRC) is on the rise. Approximately 150,000 CRC cases were diagnosed in the United States in 2019, with an estimated 50,000 fatalities due to tumor progression (Siegel et al., 2019). CRC ranks as the third most common malignancy and the second leading cause of cancer-related deaths worldwide (Keum and Giovannucci, 2019). CRC etiology is multifactorial, influenced by dietary habits, lifestyle, and the microecological environment. CRC development is a progressive process, evolving from normal mucosa through CRA to CRC. It is found that more than 90% of CRC can progress from CRA, and CRA has been recognized as a precancerous lesion of CRC. Currently, reducing CRC incidence and mortality relies predominantly on early screening, prevention, diagnosis, and treatment. The early diagnosis of CRC is mainly through colonoscopy and genetic screening. Although it has been widely used in clinics, it is expensive, and some tests may even bring certain risks to the subjects. Therefore, CRC research focuses on identifying effective, non-invasive, and affordable methods for early screening and diagnosis.

Tongue diagnosis is a crucial component of the Four Diagnoses in TCM and serves as an essential method for diagnosing diseases, monitoring illness changes, observing treatment effects, and predicting prognoses. This technique, over 3,000 years old, evaluates health by analyzing various aspects of the tongue, such as quality, color, greasiness, and thickness of the coating. While tongue diagnosis can objectively reflect internal physiological and pathological changes in the human body, traditional TCM tongue diagnosis has primarily relied on visual description rather than objective quantification (Jiang et al., 2012; Wu et al., 2005). This reliance often subjects diagnosis to variability influenced by factors like practitioners’ experience and environmental lighting, raising questions about its objectivity and repeatability. Additionally, the lack of specific quantitative standards has historically limited the advancement of diagnostic methods and technologies in Chinese medicine. However, recent advancements in digital image processing technology have significantly enhanced the objectivity of tongue diagnosis. For instance, the utilization of the Shanghai Daosheng tongue image acquisition system (DS01-B) has made tongue diagnosis more objective (Han et al., 2016). Moreover, employing the Lab color model (Qi et al., 2016) for comparative analysis introduces quantifiable metrics to tongue diagnosis, marking a significant improvement in this traditional method.

The tongue coating is a fundamental aspect of tongue diagnosis, defined as a layer on the tongue’s surface influenced by stomach qi, and holds significant value in clinical assessments of health and prognostic predictions. Modern medicine attributes the formation of tongue coating to factors such as excessive keratinization and elongation of tongue papillae, accompanied by oral bacteria, fungi, blood metabolites, saliva, exfoliated epithelial cells, and food residues (Seerangaiyan et al., 2018). Changes in tongue coating color, often turning yellow-brown or black, can occur due to pigment-producing bacteria. Abnormal tongue coatings are associated with factors like diminished immunity, poor oral hygiene, and aging.

Tongue-coating microorganisms constitute a highly diverse and specific microbial population on the back of the tongue and are a crucial part of the body’s microbial communities (Kazor et al., 2003; Dewhirst et al., 2010). This flora, predominantly comprising Bacteroides, Fusobacteria, Actinobacteria, and Firmicutes (Wilbert et al., 2020), reflects the majority of the microorganisms in the body. Previous studies have highlighted the significant role of these microorganisms in the formation of tongue coatings, suggesting their potential in disease diagnosis and treatment evaluation. The integration of microecological studies of tongue coatings into tongue diagnosis research provides biological substantiation for this traditional practice and enhances its scientific connotation.

In this study, the Shanghai Daosheng Tongue Image Acquisition System (DS01-B) was employed for tongue image analysis and processing, accompanied by microbial detection using 16S rRNA sequencing technology. This study compared the characteristics of tongue images and the microbial differences in tongue coatings among healthy individuals, CRA patients, and CRC patients. The findings substantiated tongue diagnosis as a potential and objective method for the early screening and diagnosis of CRC, offering valuable insights for its early detection.

## Materials and methods

### Study subjects

The research of this project has been registered with the Clinical Research Center of the Sixth Affiliated Hospital of Sun Yat-sen University, and ethical approval was received from the Ethics Committee of the hospital (Ethical approval number: 2021ZSLYEC-328). Informed consent was obtained from all participants.

The study’s cohort comprised patients from the anorectal surgery department of the Sixth Affiliated Hospital of Sun Yat-sen University, as well as healthy volunteers between March 2021 and June 2022. A total of 377 subjects were enrolled, including 56 healthy volunteers (41 of whom underwent microbial detection of tongue coating), 65 CRA patients (41 underwent microbial detection of tongue coating), and 256 CRC patients (59 underwent microbial detection of tongue coating). Tongue image features were captured using the DS01-B tongue information collection system, and tongue-coating microorganisms were analyzed using 16S rRNA sequencing on the Illumina MiSeq PE250 high-throughput sequencing platform.

### Covariate assessment and participant selection

Inclusion criteria: (1) All subjects were ≥ 18 years old and ≤ 85 years old. (2) Patients with CRA and CRC were diagnosed by pathology, and no surgical treatment and tumor radiotherapy and chemotherapy were performed before this test. (3) There is no serious heart, brain and other important organ diseases and liver and renal insufficiency. (4) No antibiotics or glucocorticoids were used in the 3 months before the sample collection. (5) No chronic digestive diseases (such as Crohn’s disease, ulcerative colitis, etc.) or metabolic diseases, such as obesity and diabetes. (6) No history of smoking or drinking. (7) No oral diseases, healthy oral mucosa and unrepaired dental caries. (8) complete information, able to closely cooperate with the doctor’s treatment and observation, and voluntarily participate in the experiment.

Exclusion criteria: (1) Those who do not meet the above inclusion criteria. (2) Patients with chronic diarrhea or acute diarrhea within 1 week. (3) There are signs of infection within 1 week, with the temperature rising above 37°C and WBC >10.0 × 10^9^/L or < 2.0× 10^9^/L. (4) Those who are participating in clinical trials of other drugs. (5) Pregnant and lactating women. (6) Failure to cooperate with observers according to the requirements of this study.

### The tongue image and tongue coating microbe acquisition

#### Tongue image acquisition

##### Tongue image acquisition instrument

In this study, the tongue diagnosis and measurement information acquisition system (DS01-B, product standard number: YZB/ Shanghai 5264-27-2011) produced by China Shanghai Daosheng Medical Technology Co., Ltd. has been approved by the State Food and Drug Administration. This system consists of a tongue image acquisition box and a computer software system, and the effective pixel of the acquisition device is more than 15 million.

##### Shooting method

Shoot on an empty stomach in the morning to avoid the influence of diet and drugs on the color of tongue coating. The physical examiner takes an upright position, with the mandible placed on the mandibular rest of the collection window, and the forehead pressed against the face frame and kept still. Tongue extension method: before shooting, train the subject’s tongue extension posture, that is, when extending the tongue, try to open your mouth as much as possible, fully extend the tongue out of the mouth, with the tip of the tongue down, and keep the tongue relaxed. It is not advisable to extend the tongue too hard, so that the tongue surface is flat and the tip of the tongue naturally droops. If repeated observation is needed, the subject can take a short break and repeat the tongue sticking process.

##### Tongue image observation and recording

According to the captured tongue image, the DS01-B system will automatically separate the tongue from the entire image and eliminate the background area around the tongue image to promote the recognition and extraction of the tongue image. If the DS01-B system stripping is not ideal, it can be manually adjusted. Then, by using standard values such as aspect ratio, color composition, position, color distribution of the tongue, and values of adjacent pixels, the DS01-B system automatically analyzes and pulls out the tongue image results.

#### TCM tongue diagnosis criteria and inclusion criteria

According to the standard of tongue diagnosis in Diagnostics of Traditional Chinese Medicine (Wenfeng, 2007), the tongue picture includes the characteristics of tongue quality and tongue coating, the characteristics of tongue quality include tongue color and tongue shape, and the characteristics of tongue coating include tongue color and tongue coating, as follows: ① Tongue color: light white, red, crimson, blue and so on; ② Tongue shape: it can be divided into chubby, dented, pricked and cracked; ③ Moss color: including white, yellow and gray-black; ④ Moss quality: including thick, thin, greasy, rotten, peeling and so on.

#### Quantitative parameter analysis of tongue image by lab

Lab color space calculation method of Daosheng (DS01-B) tongue image detection system was adopted. Lab color space calculation method was established on the basis of the international standard of color measurement formulated by CIE in 1931, and all colors were represented by the combination of L, A and B. Where l stands for Luminance, ranging from 0 (black) to 100 (white); A stands for red and green, positive numbers are red and negative numbers are green, ranging from −100 to +100; B stands for yellow and blue, positive numbers are yellow and negative numbers are blue, ranging from −100 to +100. In this study, the mean value of the whole tongue Lab was uniformly compared and analyzed.

#### Tongue coating microbe sample collection

Tongue coating microbial samples were collected in the morning before breakfast. All subjects were required to rinse their mouths before collecting samples. They were scraped three times at 360 degrees in different areas of the tongue and face with a sterile swab, and then another sterile swab was taken to repeat the same operation, and two swabs were collected. Soak it in that same PBS solution containing 2 mL, stir, sealing, centrifuging at 5000 r/min for 5 min, remove large residues, collecting bacteria, and immediately storing it at 80°C for further detection.

### MiSeq sequencing

DNA was extracted from the microbial samples of tongue coating of all subjects, and the V3–V4 region of 16S rRNA gene was amplified by PCR, and the library was constructed, and the samples were inspected, quantified and mixed, and then sequenced and analyzed by Illumina MiSeq PE250 high-throughput sequencing platform.

### Statistical analysis

All the data in the experiment were processed by SPASS23.0 statistical software package, and the data were statistically described by two-sided test with the test level *α* = 0.05. The quantitative data, i.e., numerical variables, are expressed by mean ± SD difference, and the comparison between groups is expressed by One-Way ANOVA, and the qualitative data, i.e., classified variables, are expressed by the number of cases (*n*). There is a statistical difference between them by chi-square test or fisher exact probability calculation, *p* < 0.05. Spearman correlation analysis was used to analyze tongue features in different pathological stages of CRC development. The tongue image feature image is made by Graphpad Prism 7.04 software, and the species accumulation curve and dilution curve are all made by R language tools.

## Results

### Participant characteristics of different populations in the development stage of CRC

A total of 377 subjects were recruited in this study, including 56 healthy subjects (44.6% male), with an age of 36.98 ± 10.63 years, a median (range) of 34 (18–59), a height of 1.65 ± 0.07 m, a weight of 63 ± 13.2 kg and a BMI of 22.91 ± 3.50. In the CRA group, there were 65 cases (male 64.6%), with age of 51.72 ± 13.47 and median (range) of 53 (18–75), height of 1.64 ± 0.76 m, weight of 63.36 10.22 and BMI of 23.32 2.65. In the CRC group, 256 cases (male 62.9%), age: 54.8 12.85/median (range): 56 (16–84), height: 1.64 ± 0.77 m, weight: 60.51 ± 10.70, BMI: 22.54 ± 3.44 (Table 1).

**Table 1:** Characteristics of study participants.

Characteristics		Healthy controls (HC)	Colorectal adenomal (CRA)	Colorectal cancer (CRC)	*p*-value
Cases (*n*)		56	65	256	NA
Sex	Male	25 (44.6%)	42 (64.6%)	161 (62.9%)	0.031
	Female	31 (55.4%)	23 (35.4%)	95 (37.1%)	
Age, years, (x̅ ± SD)		36.98 ± 10.63	51.72 ± 13.47	54.8 ± 12.85	0.000
Median (range)		34 (18–59)	53 (18–75)	56 (16–84)	
Height		1.65 ± 0.07	1.64 ± 0.76	1.64 ± 0.77	0.37
weight		63 ± 13.2	63.36 ± 10.22	60.51 ± 10.70	0.087
BMI (kg/m^2^)		22.91 ± 3.50	23.32 ± 2.65	22.54 ± 3.44	0.216

NA, not applicable due to selection criteria. *p* < 0.05, indicating statistical difference.

### Differential analysis of tongue image characteristics among different populations at different stages of CRC development

According to the routine observation points of tongue diagnosis in clinical Chinese medicine, we make a detailed qualitative analysis of tongue color, tongue shape, coating color and coating quality. The results showed that there was no significant statistical difference in tongue color among healthy group, CRA group and CRC group (*p* > 0.05), and they were Light red tongue, accounting for 69.6, 69.2 and 70.7%, respectively. In terms of tongue shape, there was no significant statistical difference among the three groups (*p* > 0.05). The healthy group was mainly “Corpulent tongue,” accounting for 62.5%, the CRA group was mainly “Cracked tongue,” accounting for 55.4%, while the CRC group was mainly “corpulent tongue,” followed by “Cracked tongue,” accounting for 52.0 and 49.6%, respectively. In terms of fur color, there was no significant statistical difference among the three groups (*p* > 0.05), but Yellow tongue coating (Y-coating) was dominant in healthy group and CRC group, accounting for 37.5 and 43.0% respectively, while Yellow and white tongue coating were dominant in CRA group, accounting for 43%. In the aspect of coating quality, we analyzed the thin coating, thick coating, greasy coating, rotten coating and peeling coating, and found that the thick coating was the main coating in the healthy group, CRA group and CRC group, with significant statistical differences among the three groups (*p* < 0.05), accounting for 75, 86.2 and 87.1% respectively, and the greasy coating among the three groups. There was also a certain difference (*p* < 0.05) among the three groups, accounting for 3.6, 13.8 and 17.2%, respectively (Table 2).

**Table 2:** Difference analysis of tongue image features.

Characteristics		HC (*n* = 56)	CRA (*n* = 65)	CRC (*n* = 256)	*p*-value
Tongue color	Light red tongue	39 (69.6%)	45 (69.2%)	181 (70.7%)	0.967
	Dark red tongue	8 (14.2%)	10 (15.4%)	44 (17.2%)	0.841
	Red tongue	0	0	1 (0.4%)	NA
	Purplish tongue	9 (16.1%)	10 (15.4%)	30 (11.7%)	0.558
Tongue shape	Corpulent tongue	35 (62.5%)	35 (53.8%)	133 (52.0%)	0.358
	Thin tongue	2 (3.6%)	3 (4.6%)	8 (3.1%)	0.851
	Moderate tongue	19 (33.9%)	27 (41.5%)	115 (44.9%)	0.315
	Teeth-printed tongue	30 (53.6%)	33 (50.8%)	123 (48.0%)	0.731
	Spotted tongue	3 (5.4%)	3 (4.6%)	8 (3.1%)	0.664
	Cracked tongue	26 (46.4%)	36 (55.4%)	127 (49.6%)	0.591
Coating color	Yellow tongue coating	21 (37.5%)	24 (36.9%)	110 (43.0%)	0.566
	White tongue coating	10 (17.9%)	5 (7.8%)	22 (8.6%)	0.088
	Yellow and white tongue coating	17 (30.4%)	28 (43%)	97 (37.9%)	0.352
	Gray-black tongue coating	8 (14.3%)	8 (12.3%)	27 (10.5%)	0.705
Coating quality	Thin tongue coating	14 (25%)	9 (13.8%)	33 (12.9%)	0.000
	Thick tongue coating	42 (75%)	56 (86.2%)	223 (87.1%)	
	Greasy tongue coating	41 (73.2%)	60 (92.3%)	231 (90.2%)	0.001
	Rotten tongue coating	1 (1.8%)	0	1 (0.4%)	0.348
	Peeling tongue coating	2 (3.6%)	9 (13.8%)	44 (17.2%)	0.032

*P < 0.05 indicates that the difference is statistically significant in comparison to the healthy group.*

### Lab quantitative analysis of tongue color and tongue coating color in different populations at different stages of CRC development

**Figure 1:**
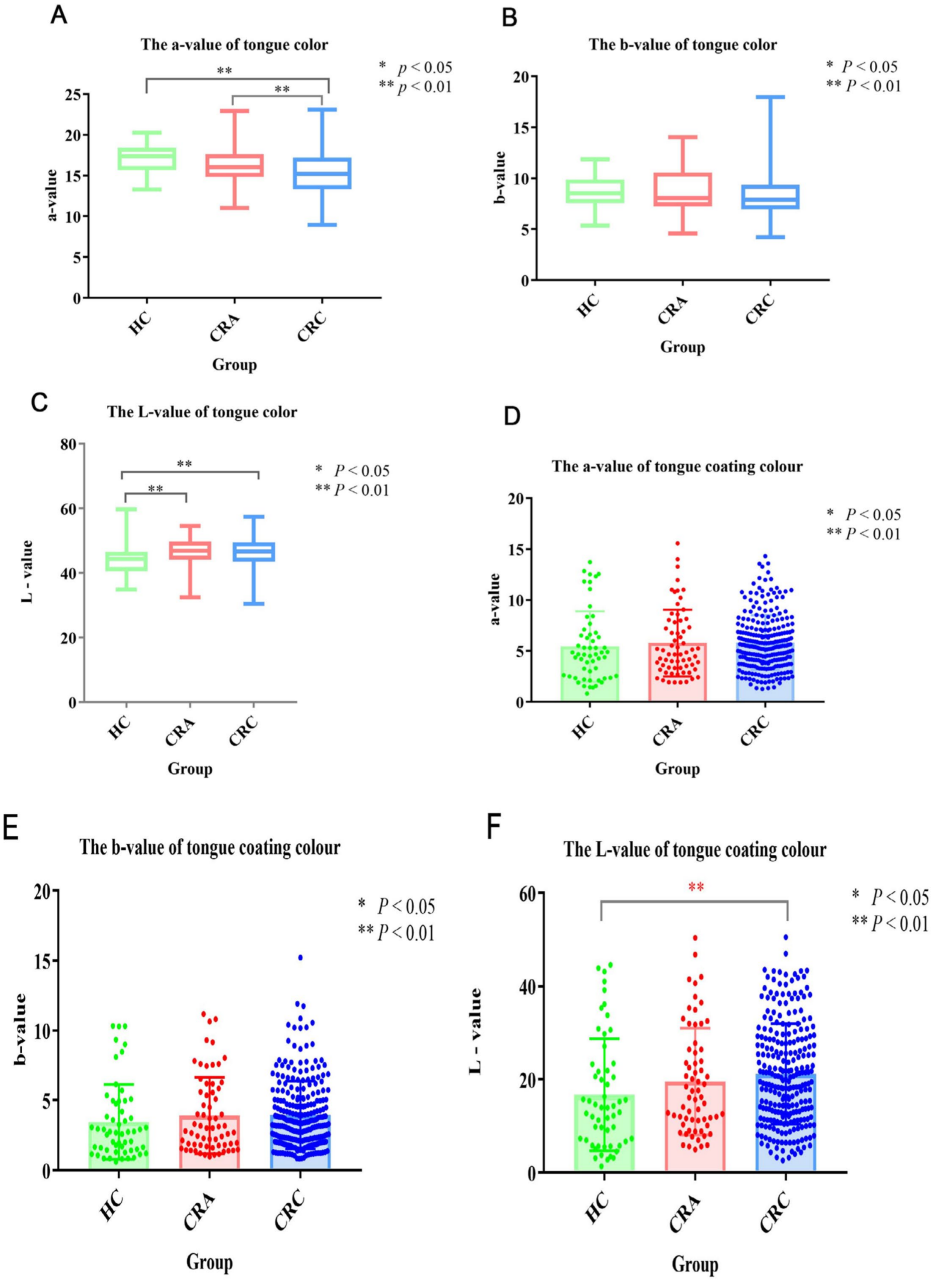
**(A)** Tongue color a-value; **(B)** Tongue color b-value; **(C)** Tongue color L-value; **(D)** Tongue coating a-value; **(E)** Tongue coating b-value; **(F)** Tongue coating L-value. * Means *p* < 0.05, ** means *p* < 0.01.

We compared and analyzed the Lab values of tongue color and fur color of people in different development stages of CRC. The results showed that the L values of tongue color in healthy group were significantly different from those in CRA group and CRC group (*p* < 0.05), which were 44.32 ± 4.67, 46.61 ± 4.66, 46.49 ± 4.62 respectively, but the L values in CRA group and CRC group were significantly different. There was no significant difference in tongue color A value between healthy group and CRA group (*p* > 0.05), but it was significantly different from CRC group (*p* < 0.01), while there were statistical differences between CRA group and CRC group (*p* < 0.05), which were 44.32 ± 4.67, 46.61 ± 4.66, 46.49 ± 4.62, respectively. B value of tongue color: there was no significant statistical difference among the three groups. In the aspect of fur color, there was no significant difference in the L value of fur color between the healthy group and the CRA group (*p* > 0.05), but there was a significant difference between the L value and the CRC group (*p* < 0.01), which were 16.71 ± 12.02, 19.53 ± 11.42, 21.18 ± 10.78, respectively. There was no significant difference in the A value and B value of moss color among the three groups (*p* > 0.05) (Figures 1A–F).

### Correlation between quantitative and qualitative analysis of tongue image features and the occurrence and development of CRC

Based on the quantitative and qualitative analysis results of tongue image characteristics, we conducted correlation analysis with the occurrence and development of colorectal cancer. The results showed that in terms of fur quality, greasy fur and stripping fur were correlated with the development and evolution of colorectal cancer, and the correlation coefficients were 0.127 and 0.121, respectively (*p* < 0.05). The L, a and b values of tongue color and the L and b values of fur color had a certain correlation with the development and evolution of colorectal cancer, and the correlation coefficients were 0.125, 0.253, 0.136, 0.159, 0.107, respectively (*p* < 0.05), and were positively correlated (Table 3).

**Table 3:** Correlation between tongue features and different stages of CRC development (Spearman analysis).

Tongue features		Correlation coefficient (R/RS)	*p*-value
Coating quality	Greasy tongue coating	0.127^*^	0.013
	Peeling tongue coating	0.121^*^	0.019
Tongue color lab value	L1	0.125^*^	0.015
	a1	0.253^**^	0.000
	b1	0.136^**^	0.008
Tongue coating lab value	L2	0.159^**^	0.002
	b2	0.107^*^	0.038

The value assigned to the healthy group was 1, the CRA group was 2, and the CRC group was 3; Greasy coating: assigned value 1, no assigned value 2. Peeled fur: assigned value 1, no, assigned value 2. * *p* < 0.05, ** *p* < 0.01.

### The characteristics of the tongue coating microbiome at different stages of colorectal cancer development

As an important part of tongue picture characteristics, in order to further explore the relationship between tongue coating microorganisms and the occurrence and development of CRC, according to the inclusion criteria and patients’ voluntary acceptance, we finally obtained 141 patients from 377 recruited subjects for tongue coating microorganism detection, including 41 healthy subjects, 41 colorectal adenoma patients, 59 colorectal cancer patients, and 16S rRNA sequencing of tongue coating microorganisms.

#### Microbial *α* diversity

**Figure 2:**
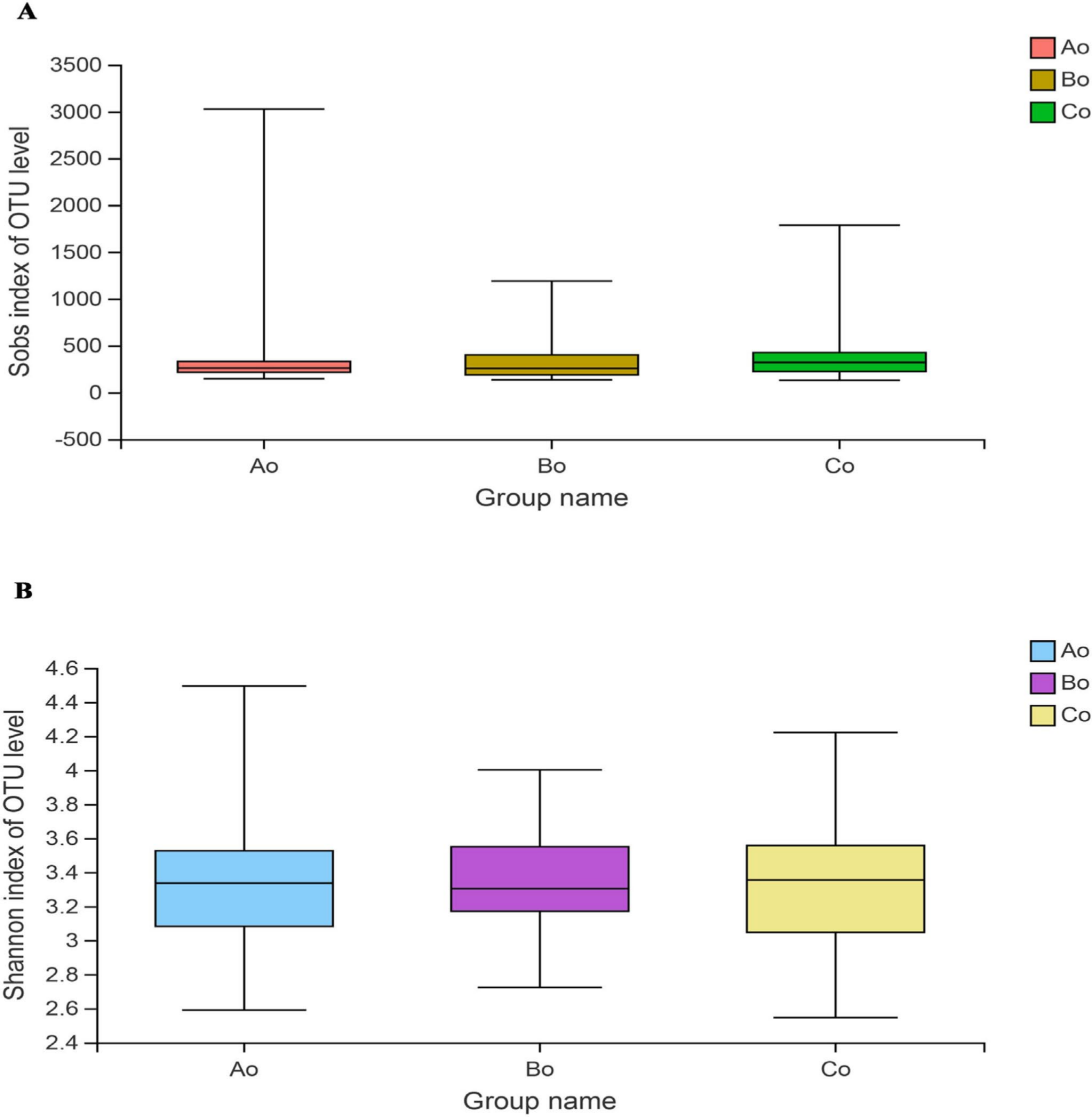
**(A)** Shows the difference of Sobs index among the three groups. **(B)** Shows the difference of shannon index among the three groups, Ao stands for healthy people, Bo stands for CRA group and Co stands for CRC group.

Through Wilcoxon Rank-Sum Test for Estimator, it was found that there was no obvious difference in the richness and diversity of microbial communities among the three groups, and the Sobs indexes of the three groups were: Ao:387.2 ± 476.95, Bo:357.59 ± 268.47, Co:399.93 ± 313.09, *p* = 0.17 > 0.05 (Figure 2A), Shannon index is: Ao:3.35 ± 0.37, Bo: 3.34 ± 0.29, Co:3.29 ± 0.36, *p* = 0.55 > 0.05 (Figure 2B).

#### Microbial species composition

**Figure 3:**
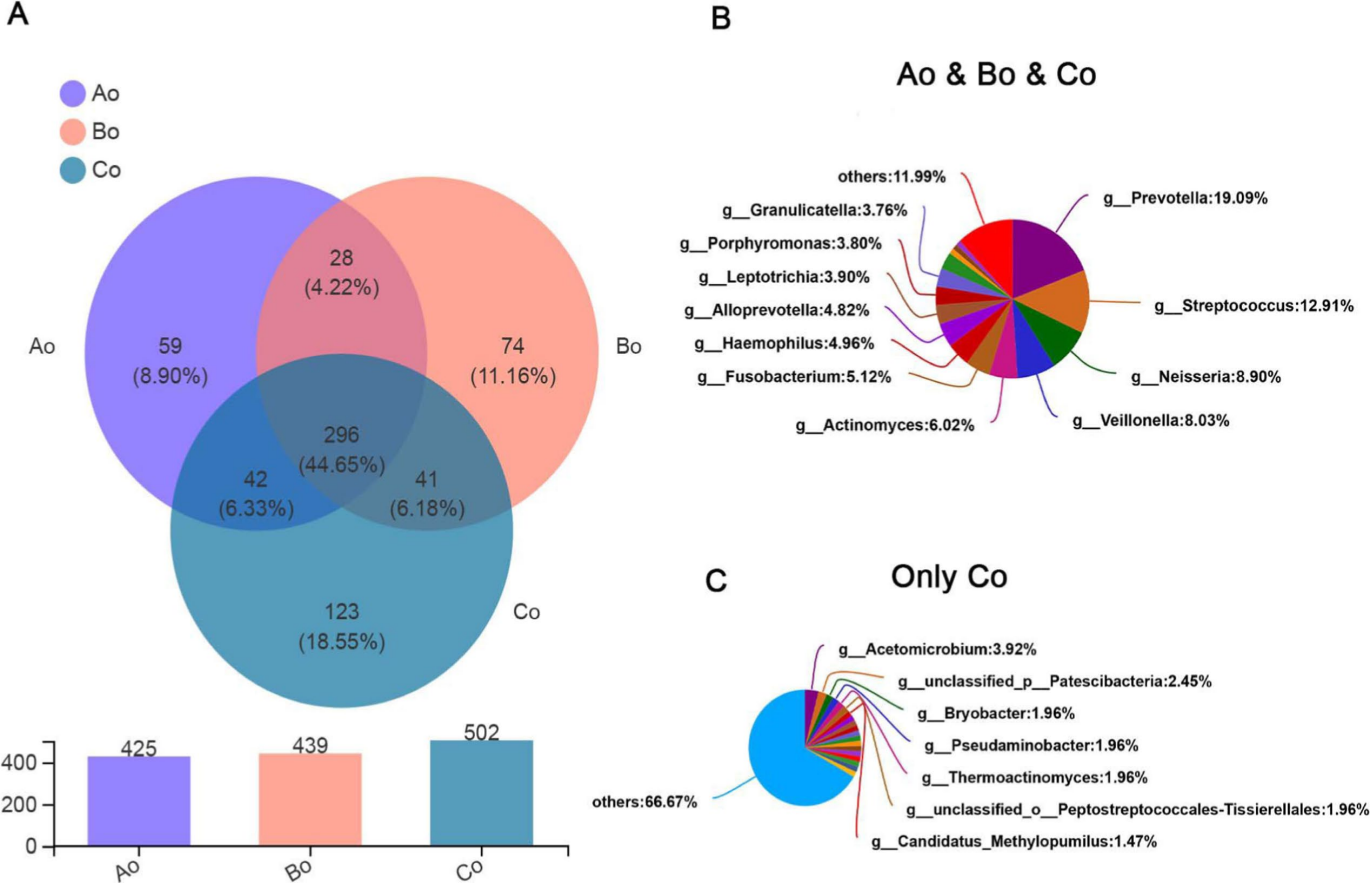
**(A)** Venn diagram, the upper figure represents different colors representing different groups of Ao, Bo, and Co, the numbers of overlapping parts represent the number of species common to multiple groups, the numbers of non-overlapping parts represent the number of species unique to the corresponding groups, and the lower figure represents the number of species in each of the three groups. **(B)** Shows the proportion of common species in three groups. **(C)** Shows the proportion of unique species in patients with CRC.

Through analysis, it was found that there were 296 species in the three groups, accounting for 44.65%, and the top5 species were mainly: g_Prevotella (19.09%), g_Streptococcus (12.91%), g_Neisseria (8.90%), g_Veillonella (8.03%), and g_Actinomyces (6.02%). Interestingly, the population of CRC group has the largest number of species, up to 502, of which the top 5 unique species are mainly: g_Acetomicrobium (3.92%), g_unclassified_p_Patescibacteria (2.45%), g_Bryobacter (1.96%), g_Pseudaminobacter (1.96%), g_Thermoac tinomyces (1.96%) (Figures 3A–C).

**Figure 4:**
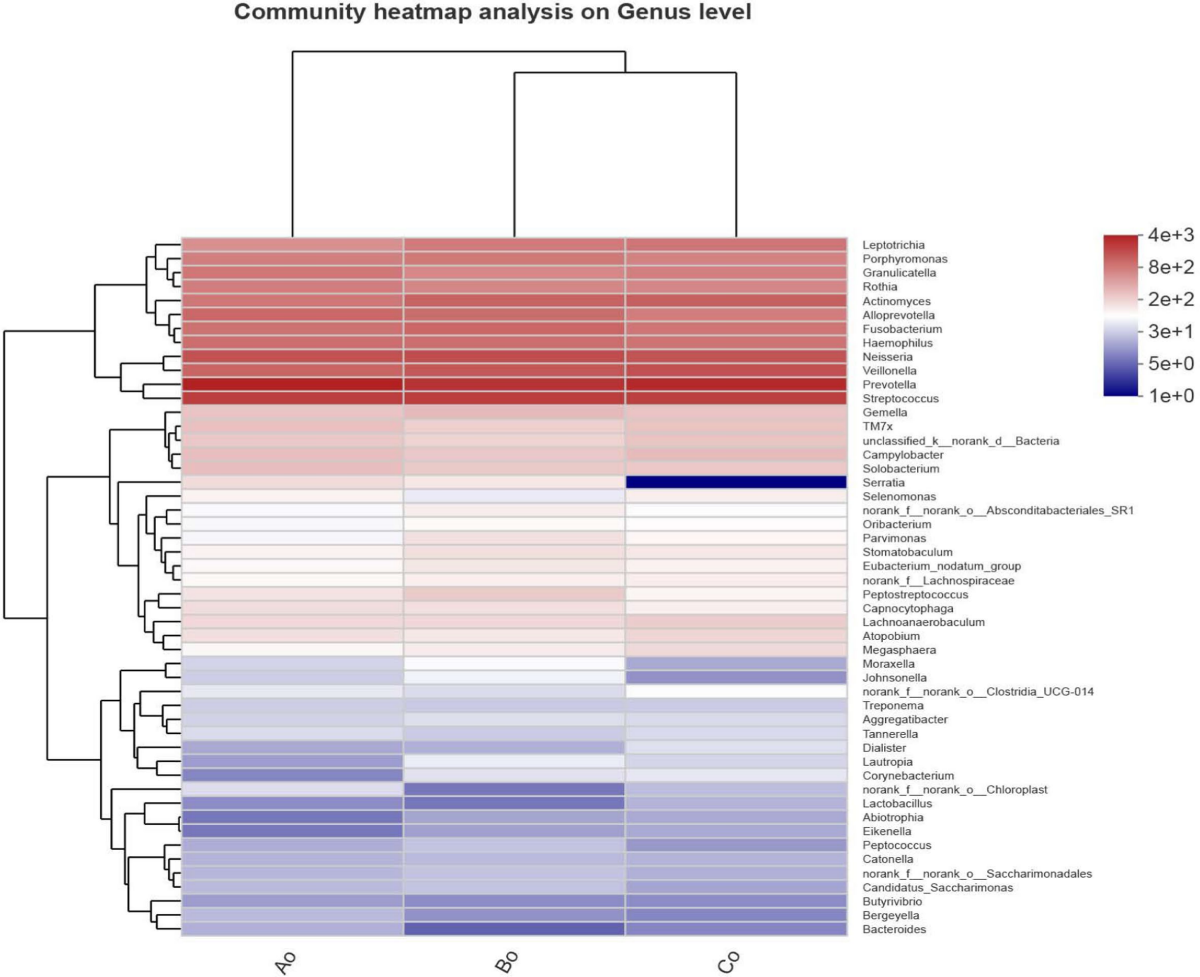
The abscissa shows the group names of Ao, Bo, and Co, and the ordinate shows the species names. The color gradient of the color block shows the abundance changes of different species in the sample, and the value represented by the color gradient is on the right.

In addition, we also made a Heatmap clustering tree analysis of the community genus level species composition of different groups of tongue coating microorganisms. The results showed that the dominant species composition of microorganisms in healthy group, adenoma group and intestinal cancer group was very similar at the genus level. Interestingly, we found that Serratia decreased significantly in colorectal cancer group, while Bacteroides decreased relatively significantly in colorectal adenoma group (Figure 4).

#### Differences in community composition among different groups

**Figure 5:**
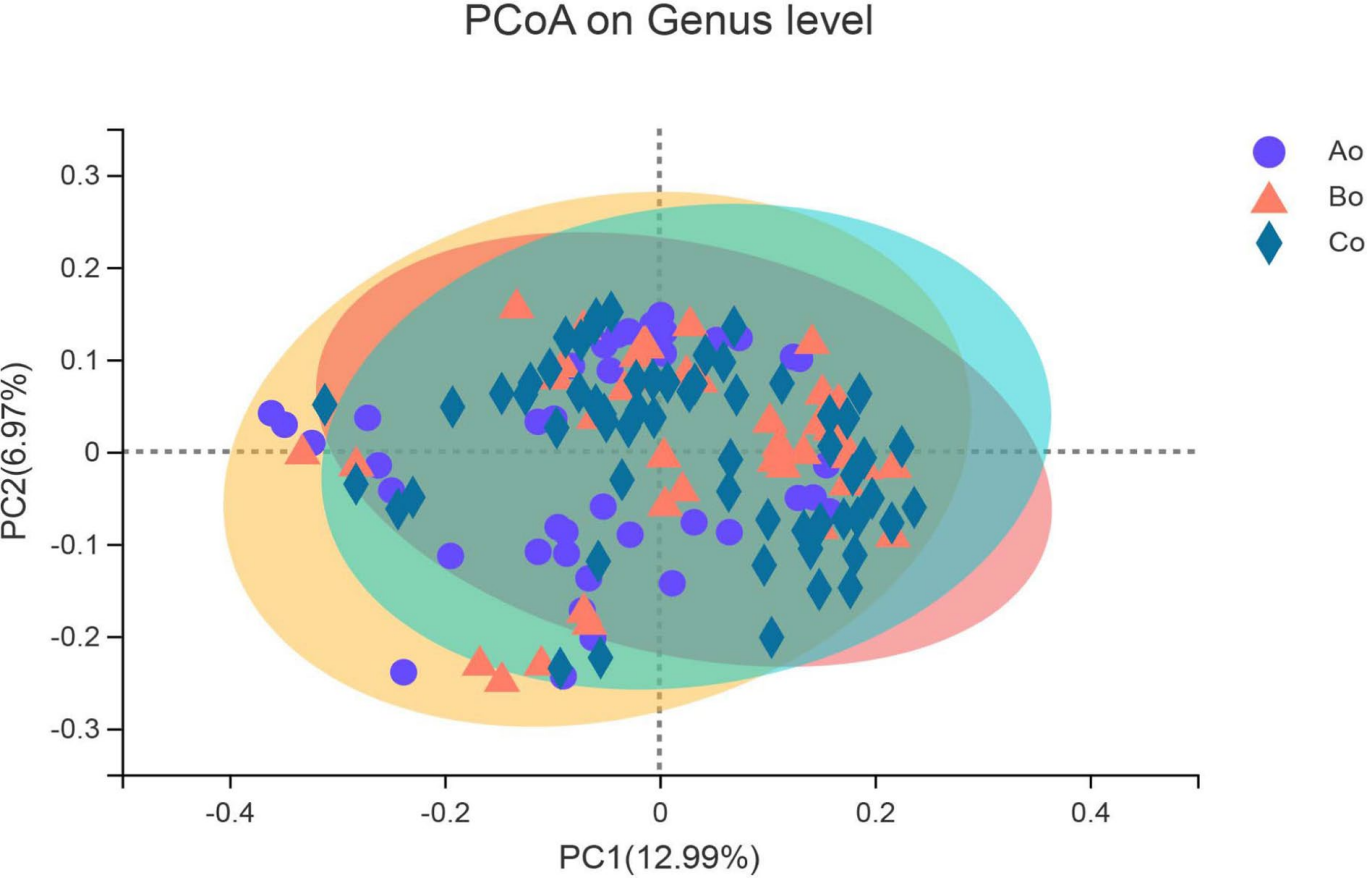
The X-axis and Y-axis represent the two selected major axes, and the percentage represents the explanatory value of the major axes to the sample composition difference; The scale of X axis and Y axis is relative distance, which has no practical significance. Points with different colors or shapes represent different groups of samples. The closer the two sample points are, the more similar the species composition of the two samples.

The composition of bacterial communities in different groups was studied by PCoA analysis of Bray-Curtis distance. The results show that there are some differences in bacterial community composition between healthy people-colorectal adenoma-colorectal cancer, and with the development of the disease, the difference in bacterial community composition is more obvious (Figure 5).

#### Analysis of microbial species differences among different groups

**Figure 6:**
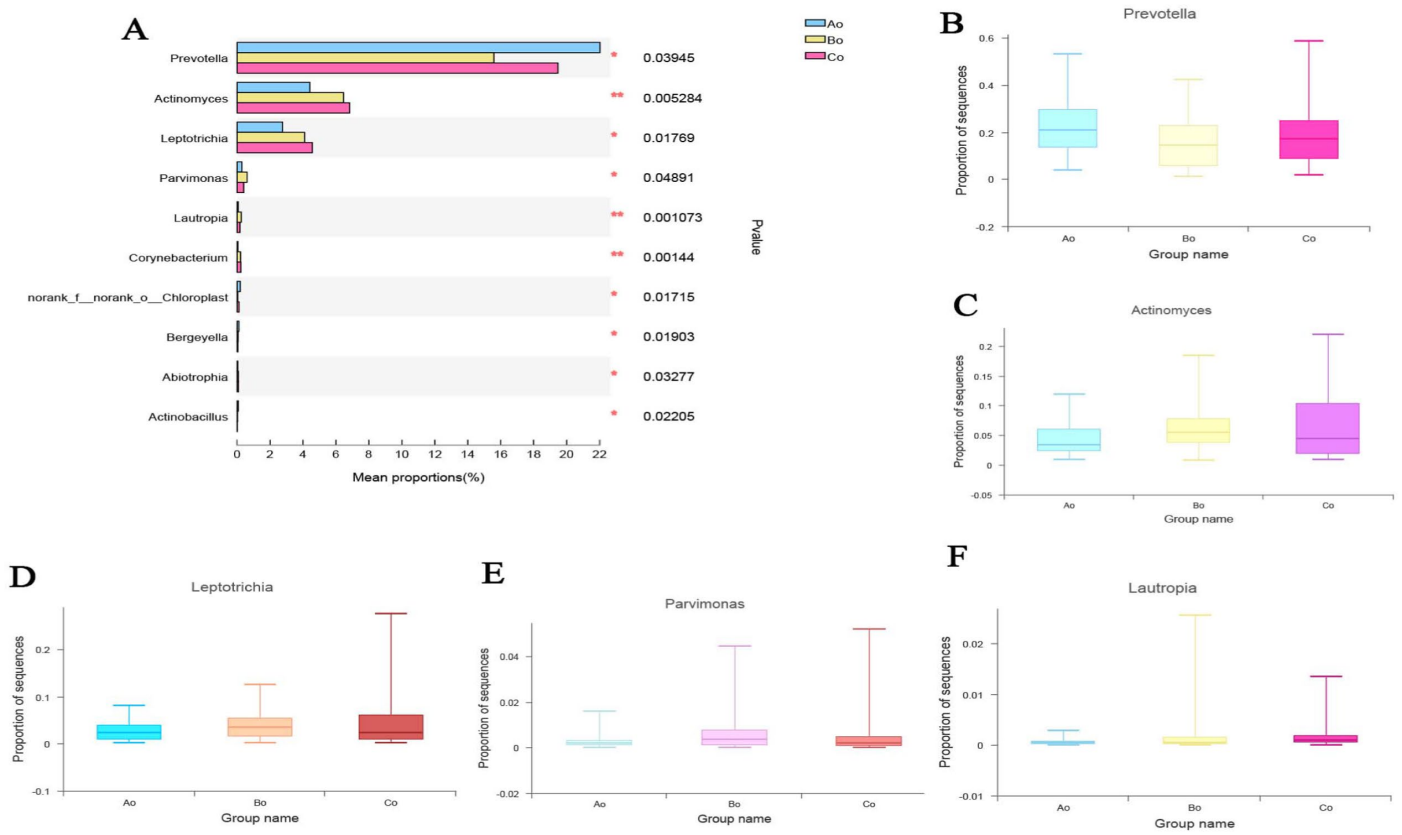
**(A)** The difference analysis of oral microorganisms in the genus level species groups of the three groups is shown. Different colors represent the groups of Ao, Bo, and Co, and the statistical method is used to sample one-way ANOVA, where the difference is expressed as * *p* < 0.05,** *p* < 0.01. **(B–F)** The specific differences of the TOP5 microbial species with obvious differences among the three groups of Ao, Bo, and Co.

Based on the data of community abundance in the sample, we used one-way ANOVA analysis to test and analyze the significant differences between three groups of horizontal species without samples. The results showed that the species composition of oral microorganisms in the three groups was significantly different at the genus level (*p* < 0.05). Top5 with obvious differences are: g_Prevotella, g_Actinomyces, g_Leptotrichia, g_Parvimonas and g_Lautropia (Figures 6A–F).

**Figure 7:**
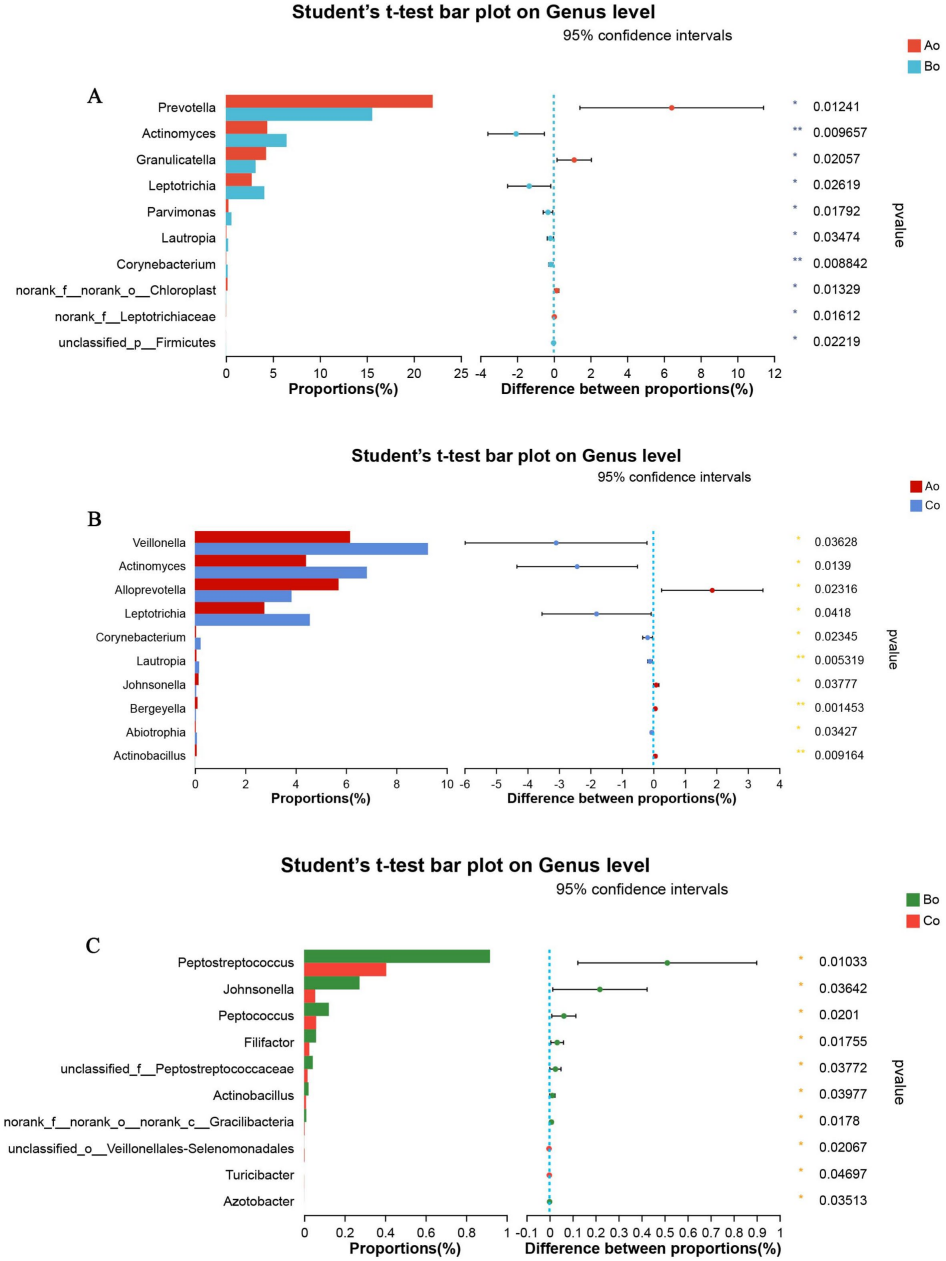
**(A)** Shows the comparative analysis of microbial species in Ao and Bo groups, where X axis represents different groups, boxes of different colors represent different groups, and Y axis represents the average relative abundance of a species in different groups. **(B)** Shows the comparative analysis of microbial species in Ao and Co groups, where X axis represents different groups, boxes of different colors represent different groups, and Y axis represents the average relative abundance of a species in different groups. **(C)** Shows the comparative analysis of microbial species in Bo and Co groups, where X axis represents different groups, boxes with different colors represent different groups, and Y axis represents the average relative abundance of a species in different groups.

In addition, in order to further explore the differences of microbial species among different groups, we compare the microbial species of the tongue coating of the three groups of Ao, Bo, and Co again. The results showed that the top 5 species with significant differences between Ao and Bo were: g_Prevotella, g_Actinomyces, g_Granulicatella, g_Leptotrichia, g_Parvimonas (Figure 7A). The TOP5 species with significant differences between Bo and Co are: g__Peptostreptococcus, g_Johnsonella, g_Peptococcus, g_Filifactor, g_unclassified_f_Peptostreptococcaceae (Figure 7B). The TOP5 species with significant differences between Ao and Co are g_Veillonella, g_Actinomyces, g_Alloprevotella, g_Leptotrichia and g_Corynebacterium (Figure 7C).

#### Discriminant analysis of LEfSe multilevel species differences

**Figure 8:**
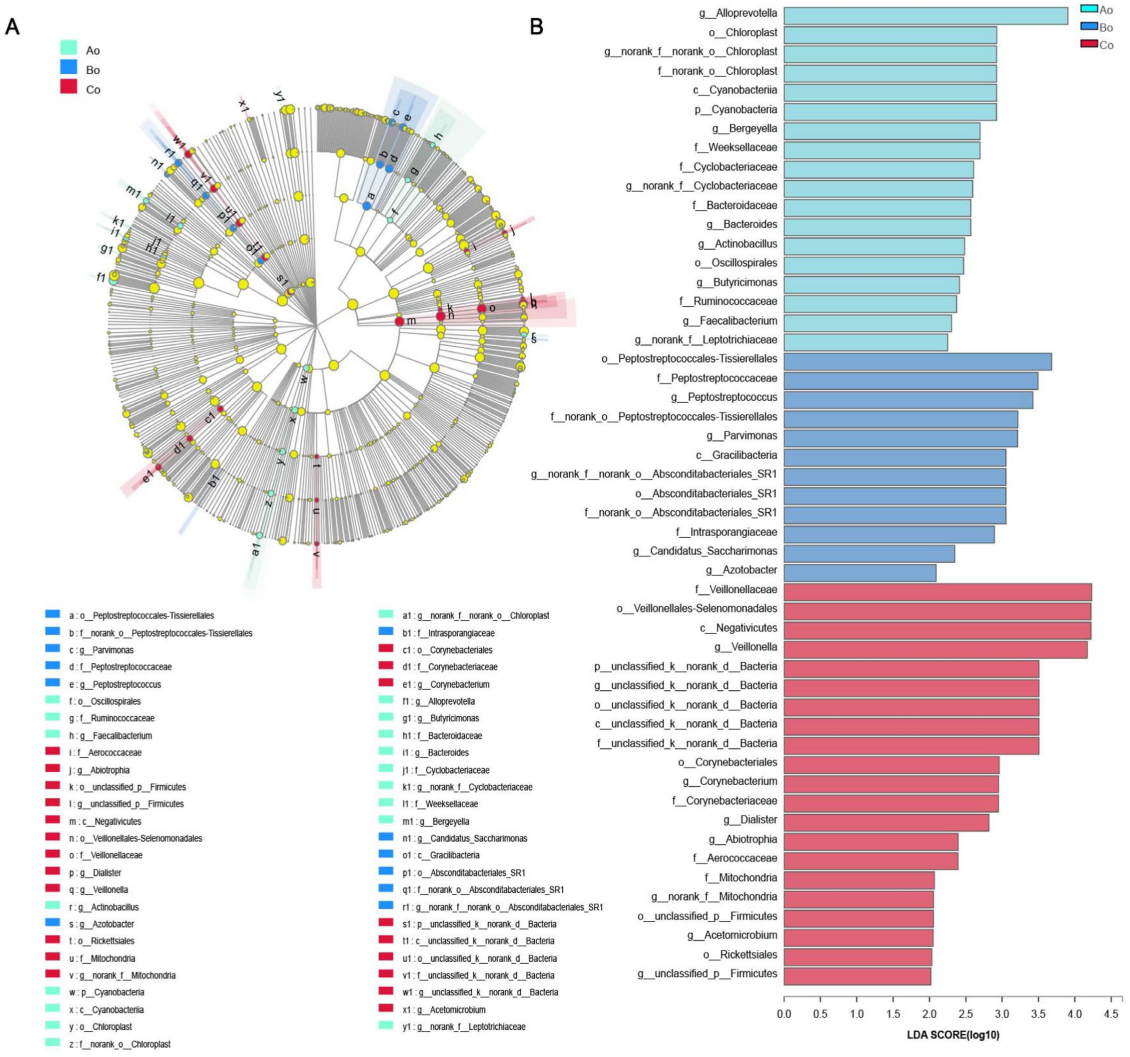
**(A)** The different color nodes represent the microbial groups that are significantly enriched in the corresponding group and have a significant effect on the difference between the groups; Light yellow nodes represent microbial groups that do not differ significantly in any of the three groups, or have no significant effect on differences between groups. There is a significant difference in the number of species on the right side of the legend position. **(B)** The LDA discriminant bar chart statistics the microbial groups with significant effects in multiple groups. The LDA score obtained through LDA analysis (linear regression analysis) indicates that the larger the LDA score, the greater the influence of species abundance on the difference effect.

By using LEfSe difference analysis, microorganisms with significant differences in Ao, Bo, and Co tongue coating microorganisms in different populations were screened from phylum level to genus level, and the LDA threshold was 2 (Figures 8A,B).

#### Single factor network analysis among the three groups

**Figure 9:**
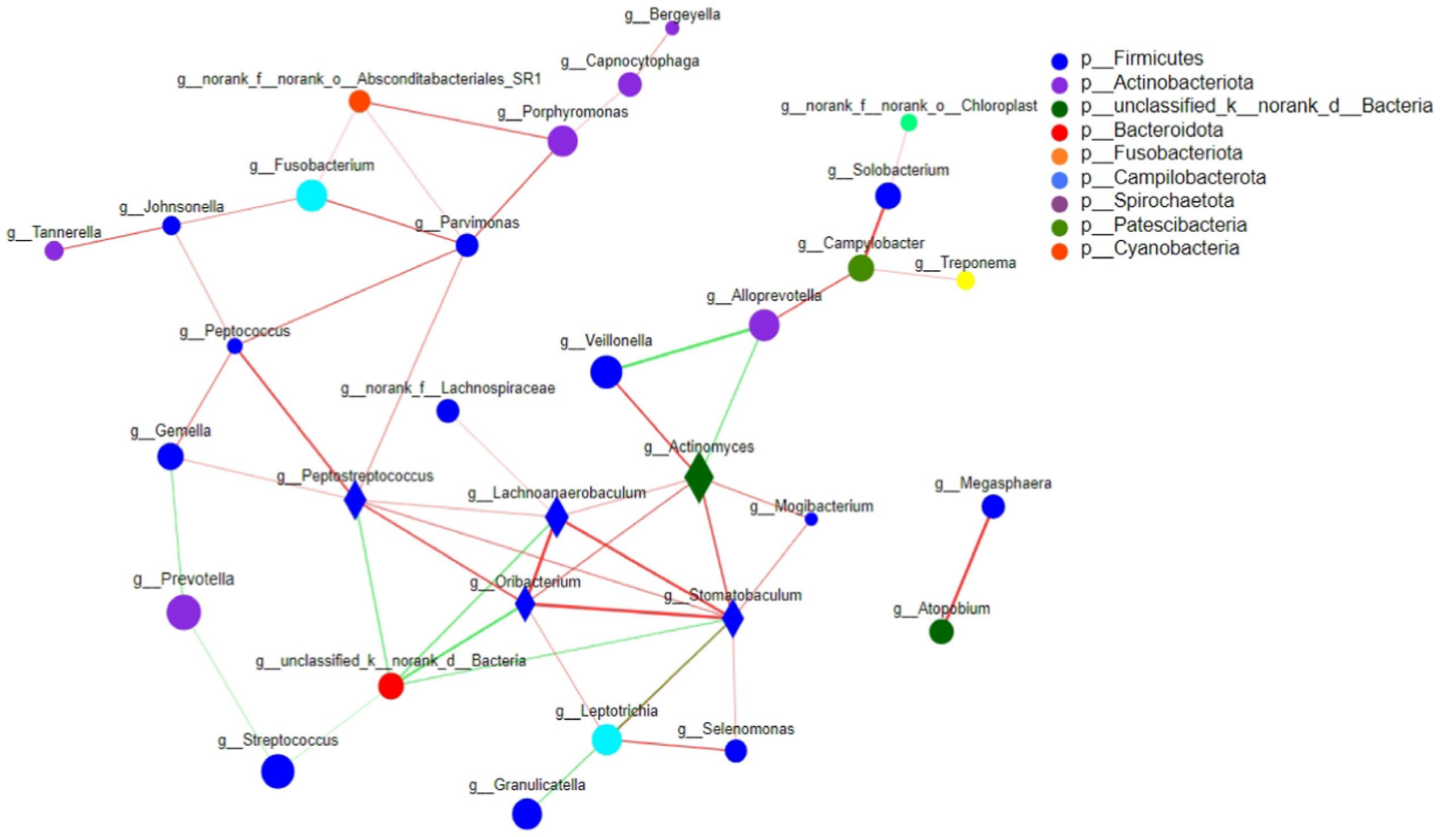
The correlation network diagram was used to analyze the relationships among the tongue coating microorganisms in the Ao, Bo, and Co groups. The horizontal correlation network map of genus revealed that there were significant interactions between different tongue coating microorganisms in different populations during the evolution of CRC. The size of nodes in the figure represents the abundance of species, and different colors represent different species. Line color Red indicates positive correlation, green indicates negative correlation; The thickness of the line indicates the size of the correlation coefficient, and the thicker the line, the higher the correlation between species. The more lines there are, the more closely related the species is to other species, and species with *p* < 0.05 are shown by default.

Based on the single factor Network analysis of the microbial composition of tongue coating in Ao, Bo, and Co groups, the top 50 species with total horizontal abundance were selected, and the correlation coefficients such as Spearman rank among species were calculated. It was found that the core species of colorectal cancer were also changing during the development and evolution. Based on the “Degree Centrality” in the network center coefficient table, We found that the core species top5 composed of tongue coating microorganisms in Ao, Bo, and Co groups are: g__Stomatobaculum, g_peptostreptococcus, g_actinomyces, G_oribacillus and g_Lachnoanaerobaculum, respectively. The core species top5 of healthy people (AO group) are:g_Peptostreptococcus, g_Leptotrichia, g__Peptococcus, g_Johnsonella and g_Gemella. The core species top5 of CRA population (Bo group) are: g_unclassified _ K_ norank _D_Bacteria, g__Peptostreptococcus, g__Stomatobaculum, g__Campylobacter and g__Alloprevotella. Patients with CRC (co group): g__Porphyromonas, g__Actinomyces, g__Alloprevotella, g__Oribacterium, g__Haemophilus. Among them, the single factor Network diagram of the microbial composition of tongue coating in Ao, Bo, and Co groups is as follows in Figure 9.

## Discussion

Tongue diagnosis is an important aspect of TCM diagnosis, which can reveal organ function and disease progress (Chen et al., 2014). It is one of the characteristics of TCM diagnosis, and it is also an important diagnostic method of China traditional medicine and Korean traditional medicine (Ko et al., 2012; Jiang et al., 2012). Tongue image is an important basis for tongue diagnosis, in which the changes of tongue quality and tongue coating are closely related to the deficiency of viscera, the rise and fall of qi and blood, and the depth of evil spirits. The changes of tongue picture characteristics such as tongue quality and tongue coating provide important information for clinical diagnosis of traditional Chinese medicine (Kim et al., 2008). Tongue coating is a kind of coating on the surface of human tongue, which can not only reflect the characteristics of pathogenic factors, but also reflect the condition of internal organs, especially the gastrointestinal tract (Sun et al., 2013).

The traditional tongue diagnosis method is mainly to observe the tongue directly, which is influenced by external factors such as light and subjective factors such as practitioners’ experience, knowledge and diagnostic skills. Due to various external and subjective factors, there are certain limitations (Kim et al., 2014). In recent years, with the development of science and technology, the objectification of tongue images has become one of the hot spots in Chinese medicine research, among which DS-01 tongue information acquisition of Chinese medicine, jointly developed by Shanghai Daosheng Medical Technology Co., Ltd. and Shanghai University of Traditional Chinese Medicine, is commonly used and has been included in the industry standard (Zhiyi et al., 2015; Yingshui, 2020; Bing et al., 2015). In DS-01 tongue diagnostic instrument, Lab color parameters are used for quantitative analysis of tongue image characteristics, among which L*a*b* color model was first developed by CIE (International Lighting Committee) (Kainuma et al., 2015). The objective information of tongue diagnosis can also be used to predict the occurrence of tumors, which is in line with the idea of “preventing diseases” in traditional Chinese medicine and is of great significance in disease prevention. As a non-invasive, simple and objective diagnostic method, tongue picture parameter detection provides new ideas and means for establishing a diagnosis and curative effect evaluation system with Chinese medicine characteristics.

In this study, according to the routine observation points of tongue diagnosis in clinical Chinese medicine, we used DS01-B tongue surface information acquisition system to make a detailed qualitative analysis of tongue image characteristics. Combined with the results of colonoscopy and pathology, we found that there was no significant statistical difference in tongue color, tongue shape and fur color among different populations in the development and evolution of colorectal cancer (*p* > 0.05), but in terms of fur quality, the three groups were mainly “thick fur,” and there were significant statistical differences among the three groups (*p* < 0.05), accounting for 75, 86.2% and 87.1, respectively. Traditional Chinese medicine believes that thick coating is caused by the steaming of stomach qi mixed with damp and turbid pathogens, so thick coating dominates the pathogen to flow into the stomach, or there is phlegm, dampness and food stagnation in it. The research results are basically consistent with the etiology and pathogenesis of colorectal cancer. There was also a statistical difference in “greasy fur” among the three groups (*p* < 0.05), accounting for 73.2, 92.3 and 90.2%, respectively. Most greasy fur is caused by dampness and turbidity, and yang is suppressed. In addition, there was a certain difference in the peeling among the three groups (*p* < 0.05), accounting for 3.6, 13.8 and 17.2%, respectively. Observing the peeling of tongue coating can measure the survival of stomach qi and stomach yin and judge the prognosis of the disease. The results show that the healthy group is significantly lower than the colorectal adenoma group and colorectal cancer group.

The color parameter model of tongue picture Lab can quantitatively analyze the tongue picture of TCM, which is one of the important methods for objective study of tongue picture of TCM. In this study, the Lab values of tongue color and fur color of people in different development stages of CRC were compared and analyzed. The results showed that the L values of tongue color and fur color were significantly different between healthy group and CRC group, which could be considered as one of the primary screening indexes to distinguish healthy people from CRC. Secondly, the A value of tongue color is significantly different between healthy group and intestinal cancer group, which can also be considered as one of the primary screening indexes for healthy people and patients with intestinal cancer. This study provides theoretical basis for quantitative study of tongue picture characteristics of CRC and early clinical screening. In addition, we further analyzed the correlation between tongue picture characteristics and the occurrence and development of CRC (that is, from healthy people—colorectal adenoma—colorectal cancer). The results showed that greasy fur and peeling fur were correlated with the development and development of colorectal cancer, and the correlation coefficients were 0.127 and 0.121, respectively, (*p* < 0.05). The L, a, b values of tongue color and the L, b values of coating color have certain correlation with the development and evolution of CRC, and the correlation coefficients are 0.125, 0.253, 0.136, 0.159, and 0.107, respectively (*p* < 0.05), and a positive correlation.

In recent years, with the development of microecology, 16S rRNA high-throughput sequencing technology has been widely used and the research of microbiology has been deepened. It has been reported from Science and Cell Host & Microbe that *Escherichia coli* and *Fusobacterium nucleatum* can induce colorectal cancer (Rubinstein et al., 2013; Arthur et al., 2012), which is the first to explore the mechanism of CRC from the perspective of intestinal bacterial infection. As the two largest microbial ecosystems of human body, the relationship between oral flora and intestinal flora has gradually attracted researchers’ attention. As an important part of oral microecology, tongue coating microecology has become the main research object of traditional Chinese medicine microecology. The complexity of the micro-ecology of the tongue is various. First, the back of the tongue is covered with filiform papillae structures and tiny gullies, which greatly increase the surface area to which bacteria attach. In addition, the warm, humid and nutritious background environment of the oral cavity also creates conditions for its growth. The number and types of microorganisms on the back of the tongue are much higher than those in other parts of the oral cavity (Nakano et al., 2002), so we can study the microorganisms on the tongue coating as the representative of oral microorganisms. Tongue coating in oral cavity, as one of the microbial communities, has a highly diverse and specific microbial population, which is an important part of the microbial community in human body (Dewhirst et al., 2010). Han et al. (2014) found that the microbial community structure on the tongue coating of CRC patients and healthy people was obviously different, and suggested that the tongue coating microbial community might become a new biomarker for patients with colorectal cancer. Ali Mohammed et al. (2021) proposed that the change of microbial community in tongue coating is related to many diseases, which can be used as a long-term monitoring and diagnosis tool for precancerous lesions or cancer cases. According to the research of Zhang et al. (2022), primary liver cancer can be accurately distinguished from healthy controls by the characteristics of tongue coating microorganism, and the tongue coating microorganism can be used as a potential noninvasive biomarker, which is suitable for long-term monitoring of primary liver cancer.

According to the Alpha diversity analysis of tongue coating microorganisms in different stages of CRC development, we found that there was no significant difference in microbial community richness (Sobs index) and diversity (shannon index) among the three groups (*p* > 0.05). Top5 of microbial species composition of tongue coating in Ao, Bo, and Co groups is consistent with the first five species of common species, mainly including g_Prevotella (19.09%), g_Streptococcus (12.91%), g_Neisseria (8.90%), g_Veillonella (8.03%) and g_Actinomyces (6.02%). However, the proportion is different, especially the proportion of g_Prevotella in CRC group is significantly lower than that in normal people (Figure 7B). It is considered that Prevost is usually regarded as a kind of bacteria related to a healthy plant-based diet, which plays the role of “probiotics” in human body, and the decrease of Prevost is related to some diseases (Ley, 2016). Colonization of Prevotella intestinalis leads to changes in microbial metabolism, which reduces the production of IL-18, thus exacerbating intestinal inflammation and may lead to systemic autoimmunity. Flemer et al. (2018) found that there is also a great difference between patients with CRC and healthy people in the genus g_Prevotella. Prevost’s bacteria not only live in the intestine, but also in the oral cavity. Among them, *Prevotella intermedia* and *Prevotella nigrescens* are related to inflammatory periodontitis, such as pregnancy gingivitis, acute necrotizing ulcerative gingivitis and adult periodontitis. The composition and metabolic activities of Przewalskii are largely regulated by diet, and they can also affect the metabolism of food. Sandberg et al. (2019) also proposed that the abundance of Enterobacteriaceae/Bacteroides might be beneficial to metabolism. Our research found that actinomycetes g__Actinomyces and ciliates g__Leptotrichia showed a gradual increase trend in the three groups of Ao, Bo, and Co, and there was a significant difference (*p* < 0.05). Yachida et al. (2019) studied the fecal metagenome and metabonomics of 616 participants in the study of intestinal microflora characteristics of patients with early colorectal cancer, and the results also showed that the number of *Actinomyces odontolyticus* increased significantly in multiple polypoid adenomas and/or mucosal cancers. At present, there is relatively little research on the genus g__Leptotrichia, which is a common colonization bacterium in human mouth and gums, and can cause local inflammation such as periodontitis and gingivitis. When immunity is low and accompanied by oral and/or gastrointestinal mucosal damage, it can also cause abdominal infection and blood flow infection. Combined with the results of this study, we consider that the occurrence of colorectal cancer may be related to the decrease of Prevotella in oral cavity and the increase of g__Actinomyces and g__Leptotrichia.

In addition, we made a single-factor Network analysis of the microbial composition of tongue coating in Ao, Bo, and Co groups, and found that the core species of tongue coating microorganisms in the three groups were: g__Stomatobaculum, g__Peptostreptococcus, g__Actinomyces, g__Oribacterium and g_Lachnoanaerobaculum, respectively. Interestingly, we found that the core species top5 of tongue coating microorganisms in patients with colorectal cancer are g__Porphyromonas, g__Actinomyces, g__Alloprevotella, g__Oribacterium, g__Haemophilus. Wang et al. (2021) found that g__Porphyromonas can promote colorectal cancer by activating inflammatory corpuscles of hematopoietic system NLRP3. Li et al. (2021) found that the abundance of Clostridium nucleatum in colorectal cancer tissues was significantly higher than that in cancer-free samples, which was significantly related to the progress of colorectal cancer. *F. nucleatum* significantly induced the expression of Cdk5 and the activation of Wnt/*β*-catenin signaling pathway. Ahn et al. (2013) found that the decrease in abundance of Fusobacterium and Porphyromonas was also considered as a high risk factor for colon cancer. In many studies, it has been found that the increase or decrease of the first three core species of tongue coating microorganisms in patients with CRC is closely related to the occurrence and development of colorectal cancer, and can even be used as specific microorganisms for early screening of colorectal cancer.

**Figure 10:**
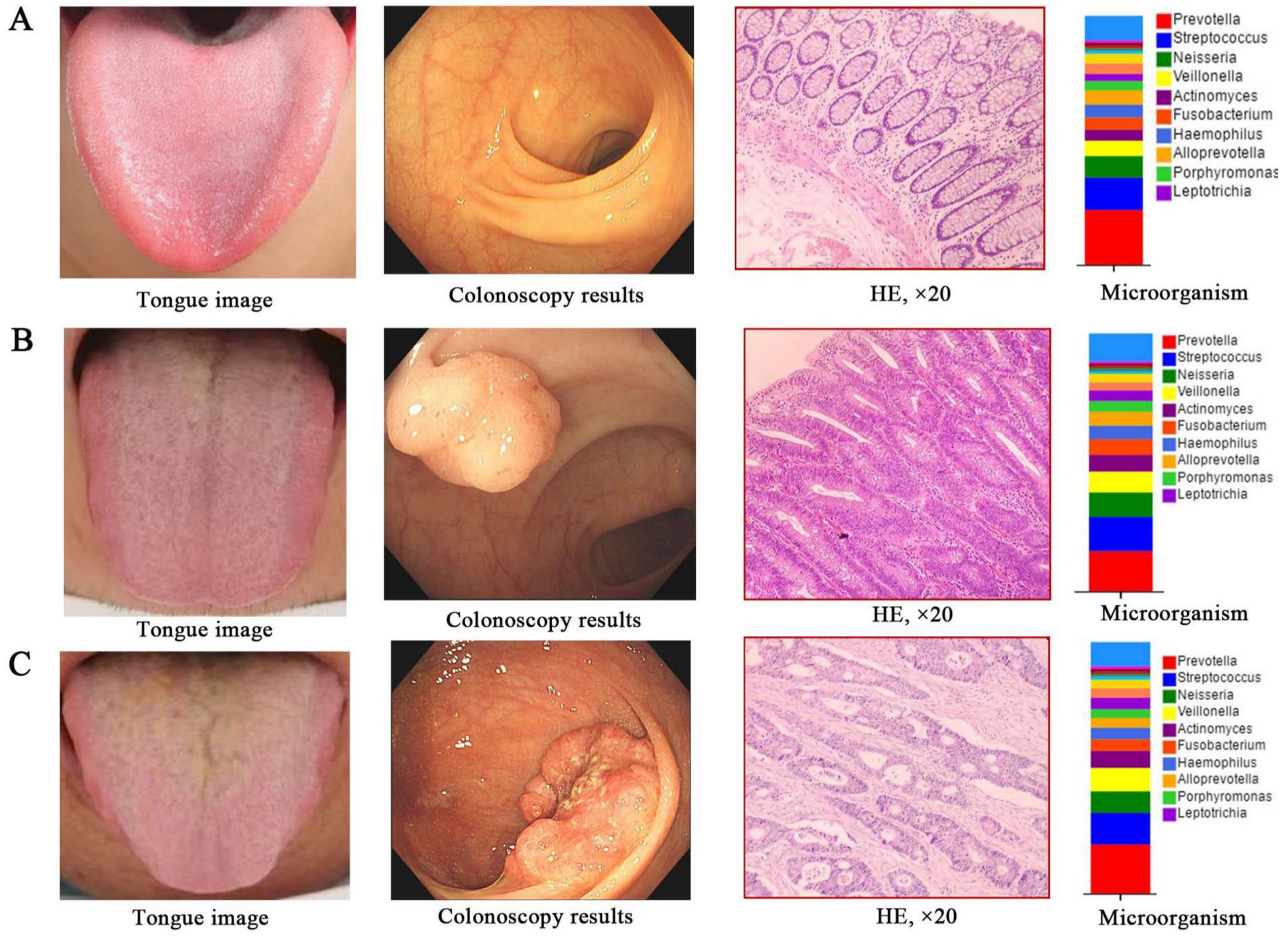
The alterations in tongue image characteristics and the shifts in tongue coating microorganisms throughout the progression of CRC. **(A)** Healthy controls; **(B)** Colorectal adenomal; **(C)** Colorectal cancer.

In summary, combined with the clinical colonoscopy and pathological results, we found that the characteristics of tongue picture and the microorganisms on tongue coating in different populations in the development stage of CRC showed obvious changes with the evolution of the disease (Figures 10A–C). The characteristics of tongue picture and the changes of microorganism on tongue coating are of great significance to the occurrence and development of CRC and even other tumors. If tongue diagnosis in traditional Chinese medicine is combined with microorganism on tongue coating as a means to screen patients with CRC, the results will be more objective and accurate, which will provide new research ideas for early screening, early diagnosis, mechanism exploration, prevention and treatment of CRC.

## Data Availability

The raw data supporting the conclusions of this article will be made available by the authors, without undue reservation.
